# Iron-Modified Blood Culture Media Allow for the Rapid Diagnosis and Isolation of the Slow-Growing Pathogen Francisella tularensis

**DOI:** 10.1128/spectrum.02415-22

**Published:** 2022-10-03

**Authors:** Efi Makdasi, Yafit Atiya-Nasagi, David Gur, Itai Glinert, Shlomo Shmaya, Elad Milrot, Theodor Chitlaru, Emanuelle Mamroud, Orly Laskar, Ofir Schuster

**Affiliations:** a Israel Institute for Biological Research, Ness-Ziona, Israel; Institut National de Santé Publique du Québec

**Keywords:** *Francisella tularensis*, tularemia, blood culture, bacterial diagnostics, iron, bacterial growth, Bactec vials

## Abstract

The life-threatening disease tularemia is caused by Francisella tularensis, an intracellular Gram-negative bacterial pathogen. Due to the high mortality rates of the disease, as well as the low respiratory infectious dose, F. tularensis is categorized as a Tier 1 bioterror agent. The identification and isolation from clinical blood cultures of F. tularensis are complicated by its slow growth. Iron was shown to be one of the limiting nutrients required for F. tularensis metabolism and growth. Bacterial growth was shown to be restricted or enhanced in the absence or addition of iron. In this study, we tested the beneficial effect of enhanced iron concentrations on expediting F. tularensis blood culture diagnostics. Accordingly, bacterial growth rates in blood cultures with or without Fe^2+^ supplementation were evaluated. Growth quantification by direct CFU counts demonstrated significant improvement of growth rates of up to 6 orders of magnitude in Fe^2+^-supplemented media compared to the corresponding nonmodified cultures. Fe^2+^ supplementation significantly shortened incubation periods for successful diagnosis and isolation of F. tularensis by up to 92 h. This was achieved in a variety of blood culture types in spite of a low initial bacterial inoculum representative of low levels of bacteremia. These improvements were demonstrated with culture of either Francisella tularensis subsp. tularensis or subsp. *holarctica* in all examined commercial blood culture types routinely used in a clinical setup. Finally, essential downstream identification assays, such as matrix-assisted laser desorption ionization–time of flight mass spectrometry (MALDI-TOF-MS), immunofluorescence, or antibiotic susceptibility tests, were not affected in the presence of Fe^2+^. To conclude, supplementing blood cultures with Fe^2+^ enables a significant shortening of incubation times for F. tularensis diagnosis, without affecting subsequent identification or isolation assays.

**IMPORTANCE** In this study, we evaluated bacterial growth rates of Francisella tularensis strains in iron (Fe)-enriched blood cultures as a means of improving and accelerating bacterial growth. The shortening of the culturing time should facilitate rapid pathogen detection and isolation, positively impacting clinical diagnosis and enabling prompt onset of efficient therapy.

## INTRODUCTION

Francisella tularensis, the causative agent of tularemia, is a Gram-negative facultative intracellular bacterium (1) considered one of the most infectious pathogenic bacteria, with high potential to cause morbidity and mortality in humans. F. tularensis has been classified as a Tier 1 biothreat agent, the route of infection and bacterial substrain determining the severity of the clinical manifestation of the disease (2). Ulceroglandular tularemia is the most common form of the disease and is usually the consequence of a bite from an arthropod vector that has previously fed on an infected animal (3). The rare pneumonic form of the disease may indicate the intentional aerosolized dissemination of F. tularensis as a bioterror agent. Early administration of effective antimicrobial therapy is essential for favorable patient outcomes (4). Thus, early detection and isolation of the pathogen are instrumental for effective treatment.

Typically, two subspecies of F. tularensis are found throughout the Northern Hemisphere, which are responsible for the majority of human infections. F. tularensis subsp. *tularensis* (type A) strains are mainly found in Canada and the United States (5), while F. tularensis subsp. *holarctica* (type B) strains are mainly found in Europe and Asia (6). F. tularensis subsp. *tularensis* can cause severe invasive diseases, such as pneumonia and bacteremia, whereas subsp. *holarctica* usually causes mild symptoms and has a low mortality rate (6, 7). The clinical significance of F. tularensis bacteremia is unknown, but it appears to occur in conjunction with more severe forms of the disease and with compromising underlying illnesses.

Diagnosing tularemia is not straightforward both due to the nonspecific nature of the initial symptoms and to the fact that F. tularensis is difficult to culture (8). Furthermore, the handling of this bacterium poses a significant risk of infection to laboratory personnel (8). In most clinical laboratories, diagnosis of tularemia relies on serological tests (which cannot be implemented earlier than 10 days from illness onset) (9), except for patients exhibiting bacteremia, in which case, tularemia may be diagnosed incidentally by the detection of F. tularensis in blood cultures (BCs). The culture of the organism is instrumental for diagnosis, antibiotic susceptibility testing, biovar characterization (10, 11), and molecular epidemiological typing (12, 13). However, direct identification of F. tularensis is limited, due to its low growth rates. Usually, the appearance of individual colonies on nonselective optimized agar plates requires 2 to 4 days of incubation, while in liquid media visible growth occurs within 3 to 7 days of incubation (14), depending the inoculum dose.

The importance of early diagnosis incentivized the development of rapid and sensitive approaches for the detection of F. tularensis directly from clinical samples. These are mainly based on immune-labeling (15–17) and genetic (9, 18, 19) assays.

Ever since blood cultures have been routinely performed for clinical diagnostics, isolation of F. tularensis from blood of infected humans has rarely been reported (20). This is possibly due to the low sensitivity of “classic” blood culturing systems and also the short duration of the bacteremia phase, early in the infection (21–23), which implies that the timing of the blood collection is critical. In recent years, with the development of more nutritious culture media, continuous-monitoring devices, and improved incubation protocols, the frequency of tularemia cases diagnosed by blood culture has increased (20, 22, 24–30). Nevertheless, prolonged incubation times are often necessary for detection, requiring in some cases as much as 12 or even 21 days for positive blood cultures to be unambiguously determined (31, 32).

Generally, the addition of nutritional supplements to accelerate bacterial growth is an effective approach. Iron was shown to be one of the limiting nutrients required for F. tularensis metabolism and growth, as established by previous studies that demonstrated restriction or enhancement of bacterial growth with the absence or addition of iron, respectively (33–35).

Commercially available ready-to-use clinical blood culturing diagnostic systems include manufacturer-provided media. However, their iron content is not standardized among manufacturers and may vary as a function of the hemin and or extracts present in the culture. In addition, an important source of iron is the analyzed peripheral blood from the sample itself; yet, this source may exhibit significant individual variations as well. Generally, the level of iron required for optimal bacterial growth is ~10^−6^ M; however, the level of free iron in mammalian tissues is typically ~10^−18^ M (36). Early studies during F. tularensis infection have suggested that iron withholding represents one of the typical host responses belonging to the innate nutritional immunity defense mechanism (37). This reduction of available iron in bacteremic blood samples combined with nonoptimal growth media can potentially limit bacterial growth in blood cultures and may result in false-negative diagnosis.

In this study, F. tularensis growth rates in iron (Fe^2+^)-enriched blood cultures were evaluated as a means of improving and accelerating bacterial growth for early diagnosis and isolation of the pathogen. To the best of our knowledge, implementation of the beneficial effect of enhanced iron concentration to expedite F. tularensis blood culture diagnostics has not been attempted previously.

## RESULTS AND DISCUSSION

### Accelerated growth rates of F. tularensis subsp. *holarctica* in Fe^2+^-supplemented blood cultures.

**FIG 1:**
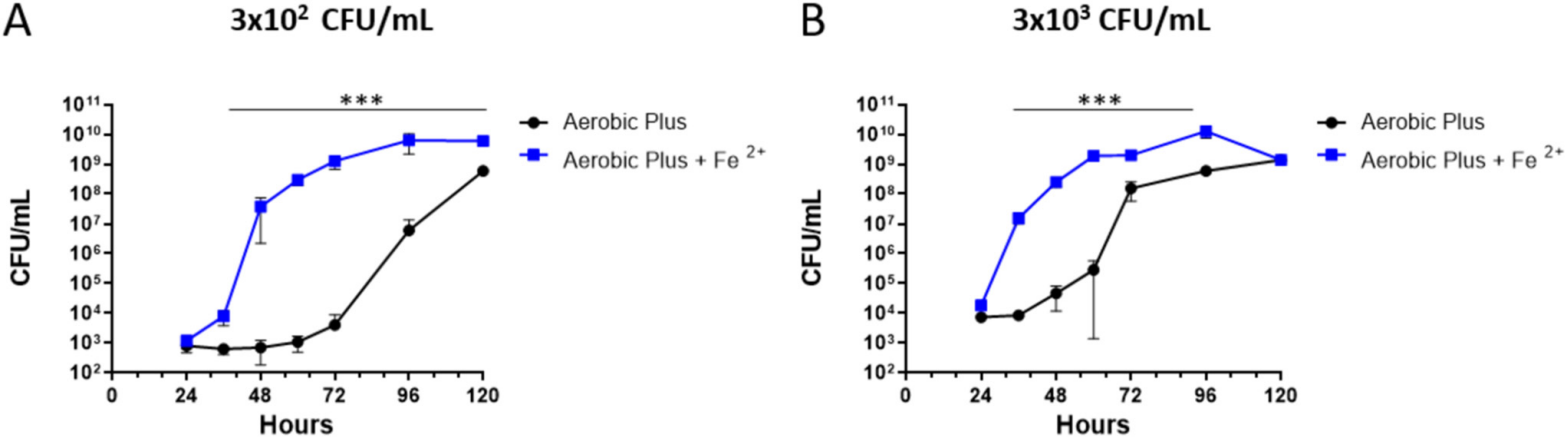
Effect of iron supplementation on the live vaccine strain (LVS) growth rate. Bactec Plus Aerobic/F culture vials containing 10 mL of naive fresh human blood were spiked with the F. tularensis subsp. *holarctica* LVS at a final concentration of (A) 300 CFU/mL or (B) 3,000 CFU/mL. Fe^2+^ (100 μM) was added to vials, and then the vials were incubated at 37°C. Bacterial growth was determined by CFU counts 0, 24, 36, 48, 60, 72, 96, and 120 h following incubation. Nonsupplemented blood cultures were used as controls. Results are averages of triplicate counts ± standard error of the mean (SEM) of results from two blood cultures for each group containing two individual blood donations. ***, *P* < 0.0001 of Fe^2+^-supplemented cultures versus nonsupplemented controls according to two-way ANOVA using Sidak’s multiple-comparison test.

In this study, the feasibility of improving bacterial growth rates in iron-supplemented blood cultures was first evaluated for the attenuated type-B F. tularensis live vaccine strain (LVS). Bacteria were spiked at two concentrations (300 and 3,000 CFU/mL) in Bactec Plus Aerobic/F culture vials supplemented with 100 μM Fe^2+^ containing 10 mL of naive fresh human blood. This Fe^2+^ concentration was previously selected after considering iron’s stability in medium versus its potential toxicity at higher concentrations (38). Vials were incubated at 37°C at 150 rpm for 120 h, and bacterial growth was determined at different time points by CFU counts. As shown in Fig. 1, increased F. tularensis growth rates were observed in blood cultures supplemented with Fe^2+^ compared to nonsupplemented media. This beneficial effect promoted a significant increase of ~6 orders of magnitude in bacterial concentrations following 48 h of incubation in the low-inoculum culture (300-CFU/mL initial concentration) (Fig. 1A). For the high-inoculum culture (3,000 CFU/mL) (Fig. 1B), an increase of ~4 orders of magnitude was observed after 32 h. These results strengthen the highly beneficial nature of Fe^2+^ addition to blood culture vials, potentially shortening F. tularensis detection time in clinical samples.

The current clinical practice of blood drawing from symptomatic adult patients for diagnostic culturing consists of collecting from two distinct sites at least two pairs of samples, each set requiring 20 mL of blood, subsequently divided equally between aerobic and anaerobic cultures (37, 39). The cultures are then incubated in a continuous monitoring system based on fluorescent detection of CO_2_ production, which serves as a reporter for potential active microbial metabolism.

Accordingly, we evaluated F. tularensis growth in various routinely employed Bactec vials in the presence or absence of additional iron. Increasing inoculums of F. tularensis (LVS, 30 to 3,000 CFU/mL) were spiked into four different Bactec blood cultures: two aerobic vials (Plus Aerobic and Standard Aerobic) and two anaerobic vials (Standard Anaerobic and Lytic Anaerobic) containing 10 mL of naive fresh human blood. The effect of Fe^2+^ supplementation in shortening the time necessary for detection was evaluated using Bactec FX40, an automated alert incubator, routinely used for clinical blood culture diagnostics. All Fe^2+^-supplemented aerobic cultures were distinguished as positive considerably earlier than nonsupplemented vials (Table 1), as expected on the basis of the above-described elevation in bacterial growth rate promoted by Fe^2+^ addition (Fig. 1). This observation was particularly significant considering that the growth rates observed at the low inoculum dose (30 CFU/mL) of F. tularensis in nonsupplemented Plus Aerobic vials were very low, virtually undetectable during the manufacturer-specified 5-day incubation protocol (Table 1), while iron-supplemented vials were detected after 76 ± 6 h. Prolonging the incubation of the Bactec FX40 for 2 additional days did not result in positive identification of the nonsupplemented cultures. Thus, Fe^2+^ supplementation dramatically shortened incubation periods for diagnosis and potential isolation of F. tularensis. Similarly, adding iron to Standard Aerobic and Plus Aerobic/F vials, inoculated with a bacterial dose of 300 CFU/mL, also resulted in significantly reduced detection times (36.7 ± 0.3 and 60.4 ± 9.6 h, respectively), compared to nonsupplemented cultures. The same effect was observed for the higher inoculum (3,000 CFU/mL), achieving a 39.7 ± 6.9-h reduction for detection in Plus Aerobic vials and 22.8 ± 5.9 h for Standard Aerobic vials. The difference between bacterial growth rates in the two Aerobic vial culture types probably stems from their formulation: Standard Aerobic vials contain hemin (see Table S1 in the supplemental material). Of note, no growth was detected in Anaerobic blood culture vials, irrespective of iron addition even at the high inoculum of F. tularensis.

**TABLE 1:** Effect of Fe^2+^ supplementation on F. tularensis early detection in spiked bacteremic blood culture using the Bactec FX40 diagnostic system

Initial inoculum	Avg ± SD time to detection (h)^*a*^								
	3 × 10^1^ CFU/mL			3 × 10^2^ CFU/mL			3 × 10^3^ CFU/mL		
	No Fe^2+^	100 μM Fe^2+^	Δ	No Fe^2+^	100 μM Fe^2+^	Δ	No Fe^2+^	100 μM Fe^2+^	Δ
Plus Aerobic	Neg	76.0 ± 6.0	>92	123 ± 7.9	62.8 ± 3.5	60.4 ± 9.6	87.3 ± 5.9	47.6 ± 1.8	39.7 ± 6.9
Standard Aerobic	NT	NT	NT	91.7 ± 2.5	55.0 ± 2.1	36.7 ± 0.3	63.8 ± 4.6	41.0 ± 1.3	22.8 ± 5.9
Standard Anaerobic	NT	NT	NT	Neg	Neg	Neg	Neg	Neg	Neg
Lytic Anaerobic	NT	NT	NT	Neg	Neg	Neg	Neg	Neg	Neg

a Neg, negative—no growth detection after 7-day incubation; N.T., not tested; Δ, the incubation time in hours was shortened for identification.

Positive cultures were confirmed by direct viable counting by plating at the time of detection. Bacterial counts were between 5 × 10^7^ and 2 × 10^8^ CFU/mL, irrespective of the original inoculum or blood culture type as previously reported (40).

### Accelerated growth rates of the virulent F. tularensis subsp. *tularensis* in Fe^2+^-supplemented blood cultures.

**FIG 2:**
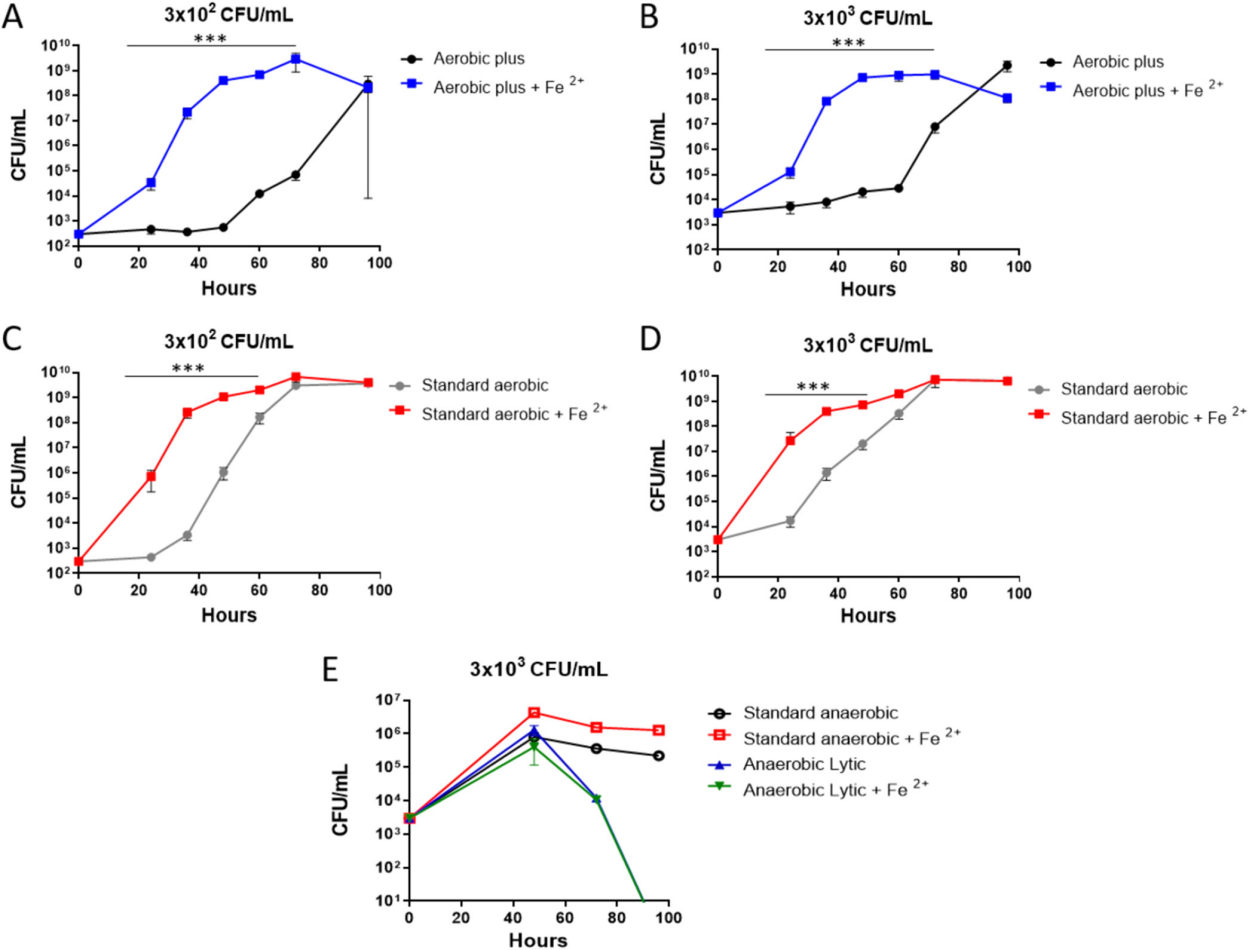
Superior bacterial growth in Fe^2+^-supplemented aerobic vials. Bactec Aerobic and Anaerobic vials (Standard Aerobic, Plus Aerobic, Standard Anaerobic, and Lytic Anaerobic) containing 10 mL of naive fresh human blood were inoculated with the virulent F. tularensis subsp. *tularensis* strain SchuS4 at (A and C) 300 and (B, D, and E) 3,000 CFU/mL. Vials were incubated at 37°C, and bacterial growth was determined by CFU counts over the course of incubation (24, 32, 48, 60, 72, and 96 h). The role of supplemented 100 μM Fe^2+^ in Plus Aerobic (A and B), Standard Aerobic (C and D), and Standard Anaerobic and Lytic Anaerobic (E) cultures was evaluated. Nonsupplemented vials were used as a control. Results are averages of triplicate counts ± SEM of results from duplicate blood cultures containing two individual blood donations in each group. ***, *P* < 0.0001, for Fe^2+^-supplemented culture versus nonsupplemented control according to two-way ANOVA using Sidak’s multiple-comparison test.

The highly beneficial effect of Fe^2+^ supplementation on bacterial growth was further demonstrated for the virulent F. tularensis subsp. *tularensis* SchuS4 strain. As depicted in Fig. 2A, a significant increase of up to 6 orders of magnitude in bacterial counts was achieved in the Plus Aerobic iron-supplemented samples within 48 h of incubation for the low inoculum tested (300 CFU/mL). For the vials containing a high initial inoculum (3,000 CFU/mL), an increase of ~5 orders of magnitude was observed (Fig. 2B). These results are in agreement with the results obtained with F. tularensis LVS (Fig. 1). Improvement of growth rate upon Fe^2+^ supplementation was also observed when SchuS4 bacteria were cultured in Standard Aerobic Bactec vials (Fig. 2C and D). In these cultures, an increase of 4 orders of magnitude in bacterial counts within 24 h of incubation was observed, irrespective of the initial inoculation concentration. This elevation reached approximately 6 orders of magnitude in bacterial counts after 32 h of incubation for the low inoculum tested (Fig. 2C).

In the absence of Fe^2+^, lower initial inoculums resulted in a delay in bacterial growth, mostly related to an elongated lag phase. Therefore, and according to the results presented in Table 1, it is conceivable to expect a more pronounced effect of Fe^2+^ supplementation in clinical, bacteremic cultures, where initial inocula are expected to be low. Generally, the bacteremia exhibited in more than 50% of the documented cases consists of less than 1 CFU/mL (41). We note that significant differences in the bacterial growth rates were observed in blood samples obtained from different donors (a total of six—two for cultures of the SchuS4 strain and four for those of LVS). These differences were more pronounced at low inocula, as represented by the high standard deviation values observed between blood samples (Fig. 2A, 100 h). Since bacteremic blood will be probably sampled after initiation of the typical nutritional immunity defense response (42), characterized by free iron reduction, it is expected that these cultures will exhibit a low growth rate. Thus, real-life clinical diagnosis should highly benefit from Fe^2+^ supplementation.

In the case of Standard Anaerobic cultures, only a marginal benefit could be attributed to Fe^2+^ supplementation and no effect at all could be detected when the SchuS4 bacteria were grown in the Lytic Anaerobic cultures, which do not support the F. tularensis growth (Fig. 2E). While elevation in bacterial growth in anaerobic condition was observed after 48 h, moderate and complete decline was observed following 96 h in Standard and Lytic vials, respectively (Fig. 2E). These results may explain the inability to achieve positive signals after 7 days in the LVS experiments (Table 1).

Finally, we verified the reliability of additional diagnostic tests, which are carried out with bacteria grown in blood cultures, for bacterial typing or for antibiotic susceptibility testing (AST) after supplementation with Fe^2+^.

**FIG 3:**
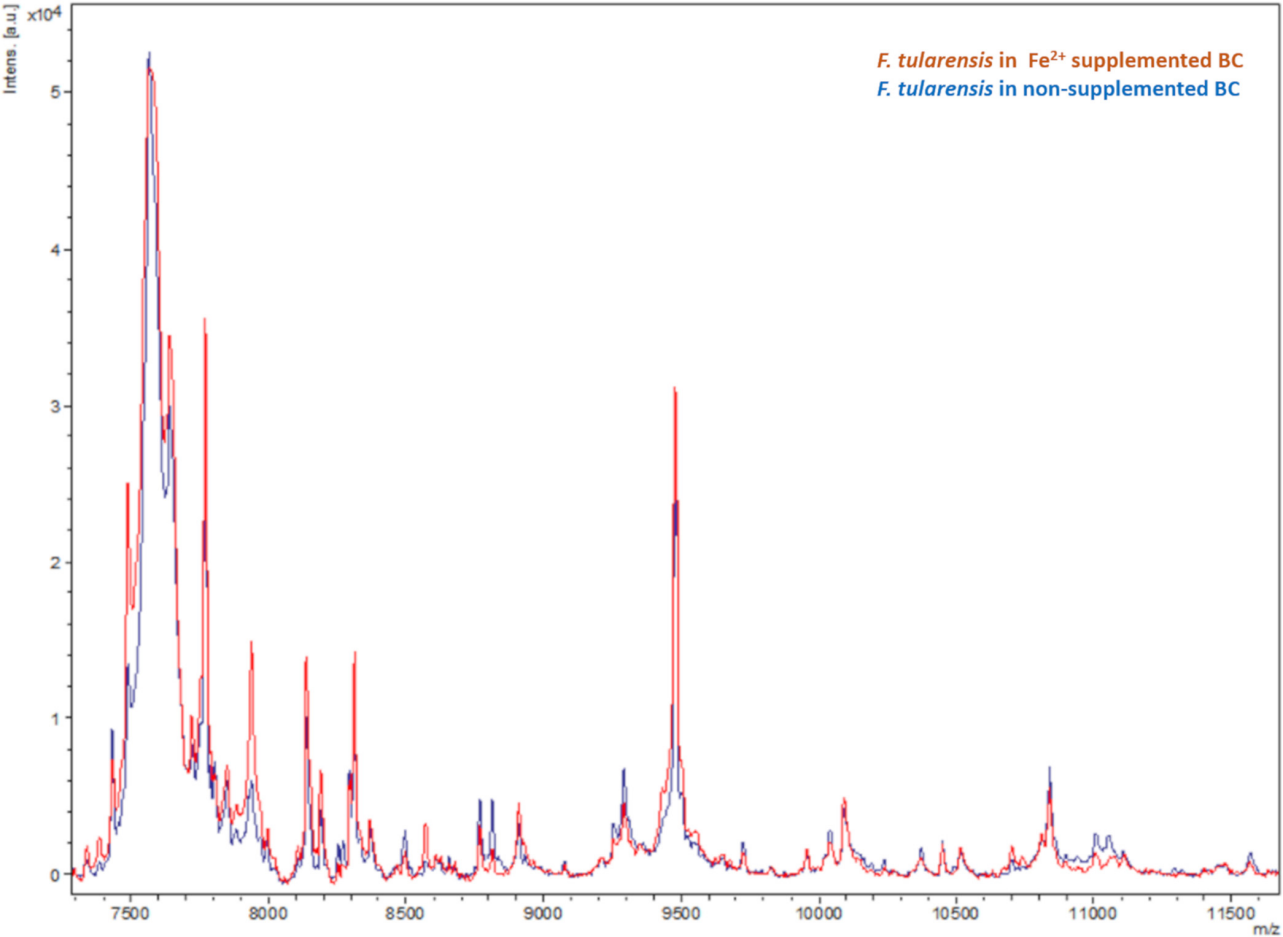
Direct detection of F. tularensis from positive blood culture by MALDI-TOF-MS. Bactec Plus Aerobic/F culture vials containing 10 mL of naive fresh human blood were spiked with LVS. Vials were supplemented with Fe^2+^ (100 μM), while nonsupplemented blood cultures (BCs) were used as a control. Shown are results from MALDI-TOF MS analysis of F. tularensis bacterial protein extraction derived directly for supplemented and nonsupplemented cultures following the Bactec FX40 incubator’s alert. The spectrum obtained from protein extracts derived from supplemented culture completely overlaps the spectrum obtained from nonsupplemented control cultures (red and blue spectra, respectively).

Rapid identification of bacteria by the matrix-assisted laser desorption ionization–time of flight mass spectrometry (MALDI-TOF MS) methodology has been extensively investigated and is frequently implemented in clinical setups as a reliable approach for identifying the bacteria present in positive blood cultures (43, 44). Accordingly, we addressed the question whether modification of the standard blood cultures by Fe^2+^ supplementation may compromise the accuracy of MALDI-TOF identification of the F. tularensis pathogen. As depicted in Fig. 3, positive F. tularensis identification (average values of 1.9) was achieved by MALDI-TOF analysis of bacterial protein extracts derived from blood cultures, regardless of Fe^2+^ content. The MS spectra obtained from both cultures completely overlapped. Thus, modification of the media allows for significantly earlier detection of growth without affecting F. tularensis MALDI-TOF identification.

**FIG 4:**
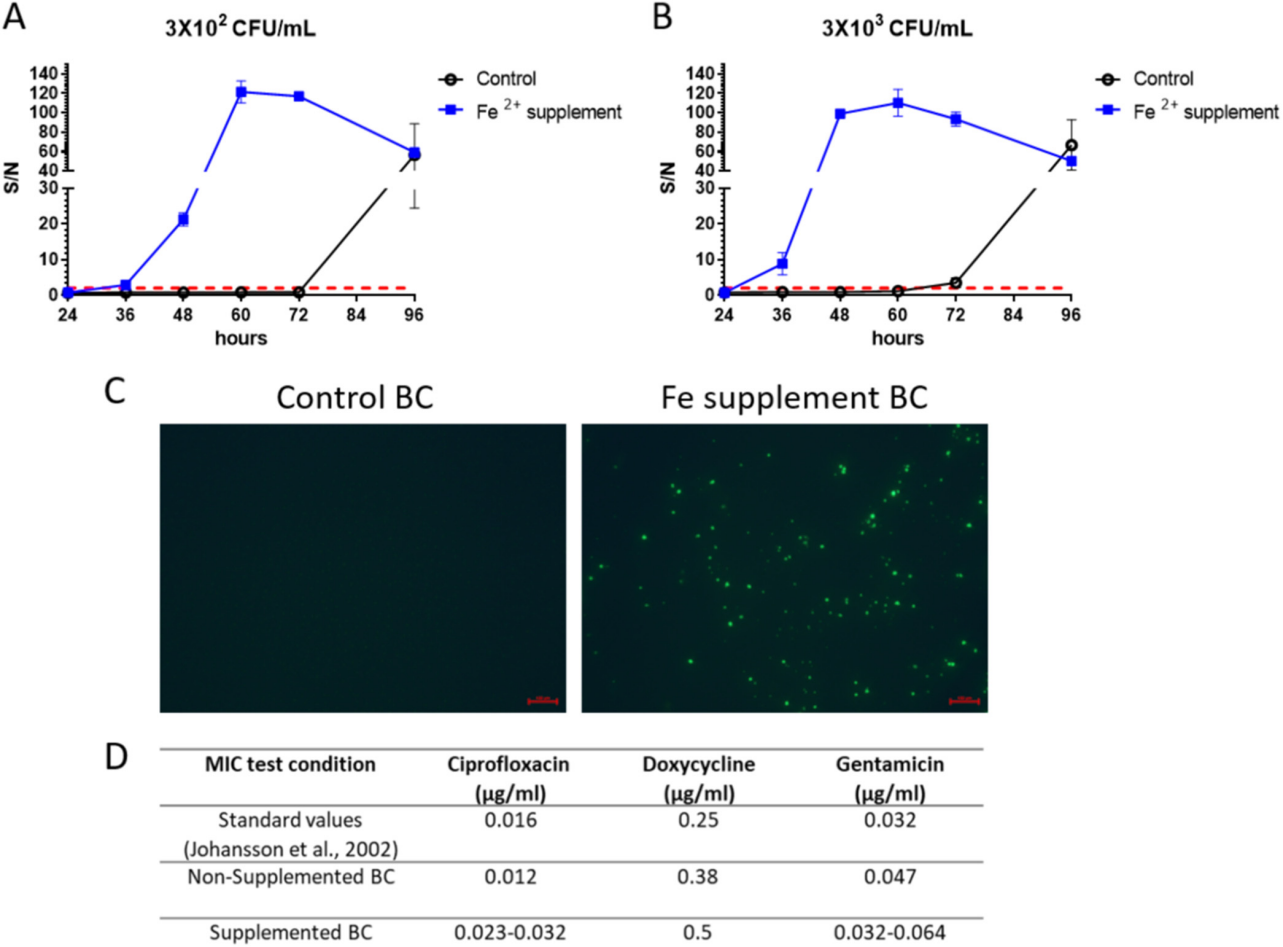
Direct immunodetection of F. tularensis from inoculated blood culture. Blood cultures (Bactec Plus Aerobic/F culture vials) supplemented with Fe^2+^ and containing 10 mL of naive fresh human blood were spiked with the SchuS4 strain at (A) 300 CFU/mL and (B) 3,000 CFU/mL. Nonsupplemented blood cultures were used as a control. Vials were incubated at 37°C, and blood cultures were sampled over the course of incubation (24, 32, 48, 60, 72, and 96 h) for the detection of soluble F. tularensis antigens by TRF. Results are presented as average ± SEM of the signal/noise (S/N) ratio from duplicate blood cultures containing two individual blood donations in each group. Positive detection was determined as an S/N ratio of >2 (red dashed line). (C) Immunofluorescence assay (IFA) using Alexa Fluor 488-conjugated anti LPS MAbs for the detection of the F. tularensis directly from blood culture following 48 h of incubation. The scale bar represents 100 μm. (D) Etest assays to determine the MIC (micrograms per milliliter) of ciprofloxacin, doxycycline, and gentamicin were performed directly from supplemented blood cultures (following 72 h of incubation) compared to nonsupplemented blood cultures and standard test values documented by Johansson et al. (45).

Since immunodiagnostics are considered reliable and specific methods for detection of tularemia (15–17), a routinely implemented immunoassay was evaluated in the modified cultures. Accordingly, supplemented and nonsupplemented Bactec Plus Aerobic blood cultures were spiked with bacteria of the SchuS4 virulent strain. Culture samples were collected in the course of 96 h of incubation. The levels of bacterial soluble antigens in the treated samples were determined by time-resolved fluorescence enzyme-linked immunosorbent assay (TRF ELISA) using polyclonal antibodies derived from rabbits immunized with the LVS strain (15). Early detection of soluble F. tularensis antigens in supplemented media was observed within 36 h, with signal/noise (S/N) ratio values of 3 or 12 for low-inoculum (300-CFU/mL) or high-inoculum (3,000-CFU/mL) cultures, respectively (Fig. 4A and B). These results are in correlation with the accelerated growth rates promoted by Fe^2+^ supplementation (Fig. 2A and B). Maximal signals (assay saturation) were achieved 48 to 60 h postinoculation of supplemented cultures compared to undetectable levels in nonsupplemented cultures. We may envisage that in the future, using supplemented cultures may represent a means by which soluble antigen detection is implemented as a stand-alone assay for rapid tularemia identification in blood culture, potentially circumventing the need of plating for CFU quantification.

Direct antilipopolysaccharide (anti-LPS) immunofluorescent staining in formaldehyde-inactivated blood culture samples was performed for F. tularensis diagnosis. The results described in Fig. 4C, establish that the sensitivity of the assay was actually improved, owing to the higher bacterial density afforded by the supplemented cultures. Finally, the supplemented blood cultures were used as a bacterial source for AST. Several antibiotics are recommended by the Centers for Disease Control and Prevention (CDC) for prophylaxis and treatment of tularemia, including bacteriostatic (e.g., doxycycline) and bactericidal (e.g., ciprofloxacin and gentamicin) drugs. Antibiogram Etest assays were performed in order to examine whether the Fe^2+^ supplementation affects the susceptibility of the bacteria to antibiotics. Therefore, the MIC of F. tularensis derived from supplemented compared to nonsupplemented blood cultures was determined. The lower density of bacteria in nonsupplemented Plus Aerobic vials following 72 h of incubation impaired the reliability of the test, while in supplemented media MIC values were accurately determined to be similar to the standard values (Fig. 4D). In addition, no significant differences in MIC values were observed in Standard Aerobic vials, irrespective of Fe^2+^ supplementation. It is conceivable that due to the higher density of the bacteria, the Etest could potentially be performed earlier. Thus, the results clearly indicate the highly beneficial value of supplementing cultures for rapid diagnosis and isolation of F. tularensis.

### Conclusions.

Tularemia, a severe life-threatening disease, is caused by F. tularensis. The low infectious doses, coupled with environmental stability, qualified this pathogen as a Tier 1 biothreat agent. Prompt administration of effective antimicrobial therapy, essential for favorable patient prognosis, requires early pathogen detection, identification, and isolation. Clinical blood samples are relevant specimens for the isolation and diagnosis of F. tularensis. Specifically, blood cultures are considered to be the “gold standard” of sepsis diagnostics. Rapid and accurate identification of the slow-growth pathogen F. tularensis using blood culture methods requires improved nutritious culture media, continuous-monitoring devices, and prolonged incubation protocols.

In order to expedite diagnostic times, supplementation of commercial, routinely used blood culture vials with external Fe^2+^ was examined for the ability to enhance F. tularensis growth rates. This study consisted of comparison of a variety of blood culturing conditions generated by supplemented or nonmodified aerobic and anaerobic protocols. The data documented in this report show a significant improvement in bacterial growth rate in Fe^2+^-supplemented media. These results were demonstrated in different blood donors, a variety of Bactec vial types, and bacterial subspecies (F. tularensis subsp. *tularensis* or subsp. *holarctica*). The study suggests that supplemented aerobic cultures represent the conditions of choice for rapid detection of the pathogen, on the basis of both bacterial growth as well as methods involving mass spectrometry, immunodetection, and identification of secreted bacterium-borne biomarkers.

In suspected tularemia cases (either upon arrival from areas of endemicity, contact with wild animals, tick bites, exclusion of more common etiologies of presenting signs, or in a suspected malicious use in bioterror scenarios) we recommend that additional supplemented aerobic vials should be included in the sampling protocol. In such cases, positive Bactec signals may prompt early downstream diagnostic assays for F. tularensis identification. In our previous study (38), we reported the accelerated bacterial growth of Yersinia pestis in blood cultures by the addition of nutritional supplements. This enabled a shortening of the doubling time, resulting in an increase of 5 orders of magnitude in bacterial loads within 24 h of incubation, allowing rapid detection and isolation of Y. pestis bacteria that grow slowly *in vitro*. Further research will establish the applicability of this metal ion supplementation for the improvement of the growth of other pathogens, possibly leading to a universal method for accelerating the pathogen’s growth and identification.

## MATERIALS AND METHODS

### F. tularensis strains.

The F. tularensis subsp. *holarctica* live vaccine strain (LVS) (ATCC 29684) and F. tularensis subsp. *tularensis* strain SchuS4 were used in this study. The study was conducted in a biosafety level 3 (BSL3) facility in accordance with the biosafety guidelines of the Israel Institute for Biological Research (IIBR).

### Culture media.

FeSO_4_ powder (Merck) was dissolved in double distilled water (DDW) and filtered (0.2-μm-pore filter), forming a 100 mM stock solution. Human blood samples were obtained from the National Blood Services, MDA, Israel, under MDA research permit 08-0290.

F. tularensis strains were grown on cysteine heart agar (CHA) plates (Becton Dickinson, France) enriched with 1% (wt/vol) hemoglobin (Becton, Dickinson, France) at 37°C for 72 h. Colonies were suspended in sterile phosphate-buffered saline (PBS) Biological Industries, Beth Haemek, Israel) and added at a defined concentration (38) into naive fresh human blood. Inoculated blood samples (10 mL/vial) were inserted into four different types of BACTEC blood culture vials (Plus Aerobic/F, Standard Aerobic/F, Standard Anaerobic/F, and Lytic Anaerobic/F (BD, Sparks, MD, USA). Blood cultures were supplemented with FeSO_4_ at a final concentration of 100 μM. Nonsupplemented blood culture vials were used as a control. The inoculated blood culture vials were then shaken at 150 rpm at 37°C in a New Brunswick Scientific C76 water bath for the indicated time periods or at 35°C in Bactec FX40 (Becton Dickinson United Kingdom) until a blood culture alert level was reached. Initial CFU counts (time 0) were determined by plating 0.1-mL blood culture samples of serial 10-fold dilutions in duplicate, while for additional time points drop plating was performed by plating 10 μL of serial 10-fold dilutions in triplicate on CHA plates. The number of CFU was determined following 48 to 72 h of incubation at 37°C.

### MIC determination.

Etest strips (bioMérieux, France) of selected antimicrobial agents (ciprofloxacin, doxycycline, and gentamicin) were applied to an inoculated CHA surface derived from spiked blood cultures with or without supplements. Each of the strips contains dried antibiotic concentration gradients that are marked with a concentration scale. The plates were incubated for 48 to 72 h at 37°C, and MIC values (micrograms per milliliter) were read directly from the strips according to the manufacturers’ instructions.

### Immunofluorescence assay for F. tularensis detection.

The immunfluorescence assay (IFA) allows visualization of the bacteria in different matrices. A 2-μL aliquot of inoculated BC with or without supplements was applied onto slides, air dried, and fixed in 100% acetone for 30 min. Alexa 488-conjugated anti-F. tularensis LPS (TL-1 monoclonal antibody) (36) was applied on the spots and incubated at 37°C for 30 min. Visualization was carried out using an Axioscop 2 fluorescence microscope (Zeiss) equipped with a ×40 magnification objective and a top-mounted camera (DS-Fi3, Nikon). Images were recorded using NIS-Elements F4.6 software.

### Detection of F. tularensis soluble antigens from inoculated blood culture by time-resolved fluorescence.

Detection of F. tularensis soluble antigens was conducted using a 3-step sandwich ELISA, based on time-resolved fluorescence (TRF) using europium lanthanide. Microtiter plates were coated with 5 μg/mL polyclonal antibodies against F. tularensis (15) overnight at 4°C. Coated wells were blocked with 2% bovine serum albumin (BSA) for 2 h at 37°C. In order to detect soluble antigens, 0.3 mL of the inoculated BC (with or without supplements) was spun at 14,000 rpm for 5 min. The supernatant was then filtered (0.2-μm pore), and a 50-μL aliquot from the sterile supernatant was applied to the microtiter plates for 30 min at 37°C. Following washing steps (with PBS containing 0.05% Tween 20), biotinylated reporter antibodies (polyclonal anti-F. tularensis) were loaded for an additional 30 min at 37°C. After an additional wash step, streptavidin-europium ab275850 (Abcam) diluted 1:1,000 was loaded for 20 min. After a final wash, enhancement solution (1244-105, DELFIA [dissociation-enhanced lanthanide fluorescence immunoassay]; PerkinElmer, Waltham, MA, USA) was added, and the resulting signal was measured using a microplate reader (excitation of 340 nm and emission of 612 nm). The results were calculated as the ratios between the signal (S) measured for each sample compared to the signal measured with the assay run against antigen-free PBS (noise [N]). S/N ratios above 2 are considered positive.

### Bacterial identification by MALDI-TOF MS.

Direct bacterial identification by matrix-assisted laser desorption ionization–time of flight mass spectrometry (MALDI-TOF MS) was carried out using a MBT Sepsityper IVD kit (Bruker Daltonics GmbH, Bremen, Germany), according to the manufacturer’s instructions. Briefly, 1 mL of blood culture was collected and transferred into a 1.5-mL tube and a 200-μL aliquot lysis buffer was added. The mix was centrifuged for 2 min at 14,000 rpm, and the pellet was washed with 1 mL washing buffer. The mixture was recentrifuged for 1 min at 14,000 rpm. The supernatant was discarded, and the pellet was dried. The sample’s proteins were extracted using the standard EX method, according to the manufacturer’s instructions. The samples were then identified by the Bruker MALDI-TOF MS instrument (Bruker Datonics, GmbH, Bremen, Germany).

### Statistical analysis.

Data were analyzed using GraphPad Prism5 software. Results are expressed as means ± standard error. Statistical significance was determined by two-way analysis of variance (ANOVA) using Sidak’s multiple-comparison test. A *P* value of ≤0.05 was considered to be significant.
