# Supplementary material for: Iron-Modified Blood Culture Media Allow for the Rapid Diagnosis and Isolation of the Slow-Growing Pathogen Francisella tularensis
Source: Microbiol Spectr. 2022 Oct 3;10(5):e02415-22. doi: 10.1128/spectrum.02415-22 (PMC9603284; doi:10.1128/spectrum.02415-22)
Supplement: Supplemental file 1 — Table S1. Download spectrum.02415-22-s0001.pdf, PDF file, 0.1 MB [file spectrum.02415-22-s0001.pdf]

Table S1. BACTEC Blood culture ingredients

	BD BACTEC™ Plus Aerobic/F	BD BACTEC™ Standard/10 Aerobic/F	BACTEC Standard Anaerobic/F	BACTEC Lytic/10 Anaerobic/F
**Processed Water**	30 ml	40 ml	40 ml	40 ml
**Soybean-Casein Digest Broth**	3%	3%	3%	2.75%
**Yeast Extract**	0.25%	0.3%	0.4%	0.2%
**Animal tissue digest**		0.01%	0.01%	0.05%
**Amino Acids**	0.05%			
**Sugar**	0.2%	Sucrose 0.1%	Dextrose 0.25%	Dextrose 0.2%
**Menadione**		0.00005%	0.00005%	0.00005%
**Hemin**		0.0005%	0.0005%	0.0005%
**Sodium Polyanetholsulfonate (SPS)**	0.05%	0.035%	0.025%	0.035%
**Vitamins**	0.025%	vitamin B6 0.001%		
**Antioxidants/Reductants**	0.005%			
**Nonionic Adsorbing Resin**	13.4%			
**Cationic Exchange Resin**	0.9%			
**Sodium Bicarbonate**		0.04%	0.04%	0.04%
**Thiols**			0.1%	0.1%
**Sodium Citrate**				0.02%
**Sodium Pyruvate**				0.01%
**Saponin**				0.26%
**Antifoaming Agent**				0.035%
